# Contextual income and incidence of disability: results of EpiFloripa Elderly Cohort

**DOI:** 10.11606/S1518-8787.2019053000659

**Published:** 2019-01-18

**Authors:** Ana Lúcia Danielewicz, Eleonora d’Orsi, Antonio Fernando Boing

**Affiliations:** I Universidade Federal de Santa Catarina. Department of Health Sciences. Araranguá, SC, Brasil; II Universidade Federal de Santa Catarina. Department of Public Health. Florianópolis, SC, Brasil

**Keywords:** Aged, Disabled Persons, Activities of Daily Living Income, Socioeconomic Factors, Independent Living, Cohort Studies, Idoso, Pessoas com Deficiência, Atividades Cotidianas, Renda, Vida Independente, Fatores Socioeconômicos, Estudos de Coortes

## Abstract

**OBJECTIVE:**

Evaluate the association between contextual income and the incidence of disability in basic and instrumental activities of daily living.

**METHODS:**

This is a cohort study, with sample of elderly individuals (n = 1,196) residing in Florianópolis, state of Santa Catarina, Brazil. The incidence of disabilities was evaluated using reports of difficulty or inability to perform six basic activities of daily living and nine instrumental activities of daily living after four years. Contextual income was obtained from the 2010 Census. We conducted multilevel logistic regression analyses with adjustment models for individual variables.

**RESULTS:**

The incidence of disability in basic activities of daily living was 15.8% (95%CI 13.8–17.9) and in instrumental activities of daily living incidence was 13.4% (95%CI 11.6–15.5). We observed significant association between contextual income and incidence of disability in basic activities of daily living. Having as reference the elderly living in the lower income tercile, those who lived in the intermediary terciles and in that of highest income had 37% (95%CI 0.41–0.96) and 21% (95%CI 0.52–1.19) lower chances of developing disability, respectively. For the incidence of disability in instrumental activities of daily living we observed no statistically significant associations.

**CONCLUSIONS:**

Contextual income influences the development of disability in basic activities of daily living in the elderly and should be the subject of actions to reduce socioeconomic inequalities and promote longevity with independence.

## RESUMO

**OBJETIVO:**

Avaliar a associação entre a renda contextual e a incidência de incapacidade nas atividades básicas e instrumentais da vida diária.

**MÉTODOS:**

Trata-se de estudo de coorte, com amostra de idosos (n = 1.196) residentes em Florianópolis, SC. As incidências de incapacidades foram avaliadas por meio do relato de dificuldade ou inabilidade para realizar seis atividades básicas da vida diária e nove atividades instrumentais da vida diária após quatro anos. A renda contextual foi obtida a partir do Censo Demográfico 2010. Foram realizadas análises de regressão logística multinível com modelos de ajuste para variáveis individuais.

**RESULTADOS:**

A incidência de incapacidade nas atividades básicas da vida diária foi de 15,8% (IC95% 13,8–17,9) e nas atividades instrumentais da vida diária de 13,4% (IC95% 11,6–15,5). Houve associação significativa entre a renda contextual e a incidência de incapacidade nas atividades básicas da vida diária. Tendo como referência os idosos residentes no tercil inferior de renda, aqueles que moravam nos tercis intermediários e no de maior renda tiveram 37% (IC95% 0,41–0,96) e 21% (IC95% 0,52–1,19) menores chances de desenvolver incapacidade, respectivamente. Para a incidência de incapacidade nas atividades instrumentais da vida diária não foram verificadas associações estatisticamente significativas.

**CONCLUSÕES:**

A renda contextual influencia no desenvolvimento de incapacidade nas atividades básicas da vida diária em idosos, devendo ser alvo de ações para redução de iniquidades socioeconômicas e promoção da longevidade com independência.

## INTRODUCTION

Demographic projections indicate that about two billion people will be aged 60 years or more in 2050. Of these, 80% will be living in middle and low income countries, which will represent a challenge to the organization of urban space, health services, and other public policies in these locations^1^. Among the main implications arising from the accelerated aging process, the increased incidence of chronic diseases and, consequently, the years spent with disabilities should be highlighted^2^.

Disability in the elderly is usually determined based on the difficulty or inability to perform daily activities independently. These activities can be classified into basic activities of daily living (BADL), related to self-care, and instrumental activities of daily living (IADL), related to the tasks of social independence^3^. Brazilian data for 2013 showed that 6.8% and 17.3% of seniors reported little difficulty to perform at least one BADL and one IADL, respectively^4^. As for incidences, 17.8% of seniors monitored over a period of three years in São Paulo developed disability in up to two activities (BADL or IADL)^5^.

According to the International Classification of Functioning, Disability, and Health (ICF)^6^, among factors that may contribute to occurrence of disability are personal factors, such as sex and age, and environmental factors, including characteristics of place of residence. In systematic review^7^, it was found that contextual factors, such as lower income of neighborhood or surroundings, are associated with higher chances of disability in BADL and IADL, in adults and in the elderly. The main hypotheses relate to the fact that lower income neighborhoods provide few opportunities for social interaction between residents^8^ ^,^ ^9^ and higher difficulties of access to health services^10^, which contributes to social isolation, illness, and consequent loss of the ability to perform activities of daily life.

However, these findings do not necessarily reflect the reality of the Brazilian context, since the samples used in the review study comprised mainly American and European subjects, whose countries present better socioeconomic characteristics than those observed in Brazil. In addition, most studies on the subject present cross-sectional design. In Brazil, we found only two studies that investigated the incidences of disabilities in activities of daily life^5^ ^,^ ^11^, and none that analyzed longitudinally the association of different contextual income strata with their occurrence.

Disability is an important marker in the health of the elderly population, not only because it reflects their worse general health condition, but also because it substantially raises their risk of mortality^12^. Therefore, findings related to the influence of modifiable risk factors on the occurrence of disabilities, such as neighborhood income, become important to contribute to actions that seek the extension of longevity with independence. Thus, this study aimed to: i) evaluate the incidences for BADL and IADL over four years; and ii) estimate the association between contextual income and incidences observed in seniors living in Florianópolis, state of Santa Catarina, Brazil.

## METHODS

This is a household, population-based study, with longitudinal design, using data from the EpiFloripa Elderly cohort, which investigates the living and health conditions in representative sample of elderly individuals residing in the urban area of the municipality of Florianópolis, state of Santa Catarina, Brazil (http://www.epifloripa.ufsc.br).

The sample of this study was composed of seniors of both sexes, aged 60 years or older, not institutionalized, participants of the baseline (2009/2010) and of the first follow-up of the cohort (2013/2014). Of the 1,705 seniors interviewed in baseline, 1,196 composed the final sample of the first follow-up, a total of 70.2% of response rate. In addition to the deaths determined in the Mortality Information System (SIM), we considered as follow-up losses all seniors not found after four attempts of interview (at least one at night and one in the weekend), who were currently hospitalized, or who had moved out of the municipality. Refusals included seniors who refused to answer even after being visited at home (Figure).

**Figure:**
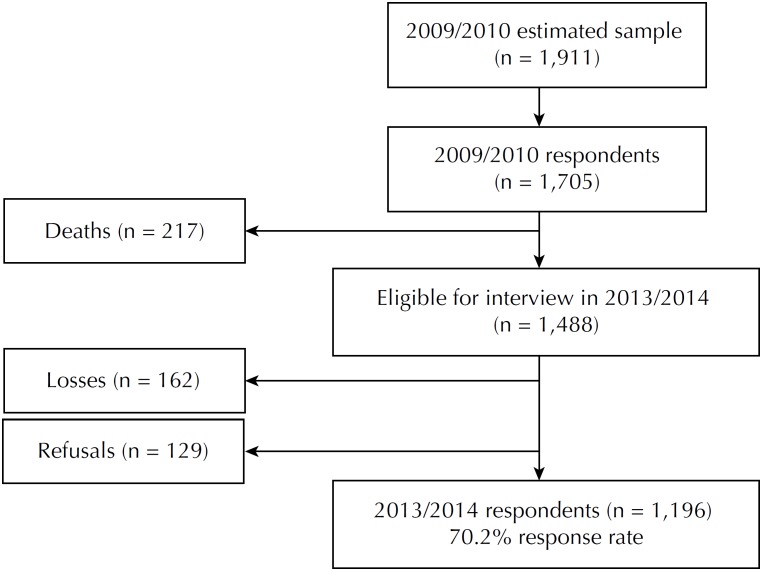
Flowchart of the 2009/2010 and 2013/2014 EpiFloripa Elderly Cohort Study. Florianópolis, state of Santa Catarina, Brazil.

Data was collected using in-person interviews in the homes of the elderly, applying the questionnaire using personal digital assistants (baseline) and netbooks (follow-up). Data consistency was assessed weekly during collection periods and quality control showed satisfactory to good consistency at the two times, with Kappa index values between 0.6 and 0.9 (baseline) and between 0.5 and 0.9 (follow-up). Further details about the methodological and cohort sampling procedures are found in another publication^13^.

The outcomes investigated in this study were the incidences of BADL/IADL disability, evaluated using the instrument Multidimensional Functional Assessment Questionnaire (BOMFAQ), validated in Brazil^14^. The questionnaire investigates the degree of difficulty (little, great, total) in performing six BADL (lie down or get out of bed, eat, walk, bathe, dress, and use bathroom) and nine IADL (take care of appearance, go up a flight of stairs, take medicines, walk close to home, shop, prepare meals, clip toenails, ride a bus or taxi, and do house cleaning). Incidence was estimated as described below:

- Incidence of disability in BADL: presence of little or great difficulty or disability to perform at least one of the six BADL investigated in the follow-up, considering seniors that presented no disability in the two domains (BADL and IADL) or that presented only in IADL in baseline.
- Incidence of disability in IADL: presence of little or great difficulty or disability to perform at least one of the nine IADL investigated in the follow-up, considering seniors that presented no disability in the two domains (BADL and IADL) or that presented only in BADL in baseline.

The main exposure was contextual income, which indicates the *per capita* income value of residents of permanent households in the census tracts sampled in the study (n = 83), estimated using the aggregated data provided by the 2010 Brazilian demographic Census^15^ and subsequently categorized into terciles. Individual adjustment variables were self-reported during the follow-up and included: sex (female or male), age (60–69 years, 70–79 years; 80 years or more), equalized *per capita* household income (estimated by dividing the mean household income by the square root of the number of residents, categorized into terciles), and time of residence in the neighborhood (0–4 years, 5–9 years; 10 years or more).

Analyses employed the statistical software Stata version 14.0. We described the incidence of the outcomes and their confidence intervals (95%CI) according to the exposure variable and individual adjustment variable. Data analysis employed Multilevel Logistic Regression. First, we tested the null model (without exposure variables) and then we included the other variables according to three models: model 1, only with the census sector *per capita* income variable; model 2, adjusted for sex and age; and model 3, adjusted for individual *per capita* income.

We estimated the values for odds ratio (OR) and the respective 95%CI of the associations, considering individual variables as first level of analysis and census tracts as second level. For each model we estimated the intraclass correlation coefficient (ICC), which provides estimates of the total variance of the outcome that can be attributed to the differences between census tracts. ICC value was defined by the following formula: Vn / (Vn + Vi); where Vn represents variance at contextual level and Vi represents variance at individual level, which has fixed value and equal to π^2^/3 (Merlo et al., 2005)^16^. All analyses considered the updating of the home addresses of the elderly respondents during follow-up and those who moved to census tracts other than those sampled in the study (n = 75) were not included in the logistic models.

The EpiFloripa Elderly study was approved by the Research Ethics Committee of the Universidade Federal de Santa Catarina (Opinion 352/2008 in the baseline) and by the Certificate of Submittal to Ethics Assessment (CAAE 16731313.0.0000.0121 in the follow-up). All respondents signed an informed consent form in the two phases of the study.

## RESULTS

The sample effectively analyzed comprised 1,196 seniors who answered questions about the BADL and IADL at both phases of the cohort study. Mean age of follow-up participants was 73.9 years (standard deviation of 7.2 years), with higher proportion of female (65.0%). We observed significant follow-up losses in the sample only for the age group variable (p < 0.001), with higher proportion of deaths among seniors aged 80 years or more (38.6%). For the other socioeconomic variables analyzed, sex and individual *per capita* income, losses were equivalent between the categories analyzed.

The incidences of disability were 15.8% (95%CI 13.8–17.9) in BADL and 13.4% (95%CI 11.6–15.5) in IADL after the four-year follow-up period. For females, we observed slightly higher incidences in both domains (BADL and IADL). As for age group, the highest incidences were observed in BADL for seniors aged 80 years or more and in IADL for those aged 70–79 years. Seniors belonging to the lower individual *per capita* income tercile showed higher incidence of disability in BADL, while in IADL no difference was observed between the lower and the higher tercile (Table 1).

**Table 1:** Description of individual and contextual characteristics according to incidence of disability in basic activities of daily life (BADL) and instrumental activities of daily life (IADL). 2009/2010 and 2013/2014 EpiFloripa Elderly study, Florianópolis, state of Santa Catarina, Brazil.

Characteristics		Sample 2009/2010	Sample 2013/2014	BADL Incidence	IADL Incidence
		n (%)	n (%)	% (95%CI)	% (95%CI)
Individual					
Age (years)					
	60–69	850 (49,9)	412 (34,4)	12,6 (9,7–16,2)	13,9 (10,9–17,6)
	70–79	616 (36,1)	509 (42,5)	15,1 (12,2–18,5)	15,2 (12,3–18,6)
	≥ 80	238 (14,0)	276 (23,1)	21,7 (17,2–27,0)	9,4 (6,5–13,3)
Sex					
	Male	616 (36,1)	419 (35,0)	13,1 (10,2–16,8)	12,9 (10,0–16,5)
	Female	1089 (63,9)	778 (65,0)	17,2 (14,7–20,0)	13,6 (11,4–16,2)
Equalized *per capita* income (terciles)					
	1^st^ tercile	553 (33,3)	401 (33,5)	20,2 (16,5–24,4)	13,5 (10,5–17,3)
	2^nd^ tercile	553 (33,3)	402 (33,6)	13,9 (10,8–17,7)	12,9 (10,0–16,6)
	3^rd^ tercile	553 (33,3)	393 (32,9)	13,2 (10,2–17,0)	13,7 (10,7–17,6)
Contextual (census sectors n = 83)					
Contextual *per capita* income (terciles in BRL)					
	1^st^ tercile (< 1,101)	235 (19,7)		19,2 (15,7–23,3)	14,9 (11,7–18,7)
	2^nd^ tercile (1,101–1,864)	260 (21,7)		12,9 (9,8–16,6)	10,7 (8,0–14,3)
	3^rd^ tercile (> 1,864)	214 (17,9)		15,1 (11,9–18,9)	14,4 (11,3–18,2)

The contextual *per capita* income median was BRL 1,291 (interquartile range of BRL 1,096) ranging from BRL 383 to BRL 5,046; incidence of disability for both BADL and IADL were higher in the lower terciles of the distribution (Table 1). In comparison between the groups, the elderly who acquired disability in the follow-up (in both domains) resided in census tracts with lower per-capita income median than those that did not develop disability.

Table 2 presents results of Multilevel Logistic Regression for incidence of disability in BADL. Both in the raw analysis (model 1) and in the analyses adjusted for individual-level variables (models 2 and 3), we observed significant associations between the intermediate contextual *per capita* income tercile and the incidence of the outcome. The chance of developing disability in BADL was 37% lower among seniors residing in sectors with intermediate *per capita* income tercile than those living in the lower tercile. And, although not significant, we observed that the elderly residing in the high *per capita* income tercile also presented lower chances (21%) of developing disability in BADL than those in the lower tercile.

**Table 2:** Multilevel logistic regression models for association between contextual income and incidence of disability in basic activities of daily living (BADL). 2009/2010 and 2013/2014 EpiFloripa Elderly study, Florianópolis, state of Santa Catarina, Brazil.

Variable		Empty model	Model 1	Model 2	Model 3
		OR (95%CI)	OR (95%CI)	OR (95%CI)	OR (95%CI)
Fixed effects					
	Intercept	0,18 (0,16–0,22)	0,24 (0,18–0,30)	0,15 (0,09–0,23)	0,32 (0,13–0,77)
Contextual level					
Contextual *per capita* income (terciles in BRL)					
	< 1,101		1,00	1,00	1,00
	1,101–1,864		0,60 (0,40–0,91)*	0,59 (0,39–0,90)*	0,63 (0,41–0,96)*
	> 1,864		0,76 (0,52–1,12)	0,72 (0,49–1,07)	0,79 (0,52–1,19)
Individual level					
Age (years)					
	60–69			1,00	1,00
	70–79			1,24 (0,83–1,85)	1,25 (0,83–1,85)
	≥ 80			1,92 (1,24–2,98)*	1,88 (1,18–2,87)*
Sex					
	Male			1,00	1,00
	Female			1,34 (0,93–1,92)	1,30 (0,92–1,88)
Equalized *per capita* income (terciles in BRL)					
	< 1,281.42				1,00
	1,281.72–3,000				0,68 (0,45–1,02)
	> 3,000				0,71 (0,46–1,09)
Random effects					
	ICC (%)	0,60	0,00	0,00	0,00

ICC: intraclass correlation coefficient

* p < 0.05

Table 3 shows results observed for incidence of disability in IADL. In this outcome, we observed no statistically significant associations between the contextual *per capita* income terciles. We also found that, for both outcomes (BADL and IADL), ICC values had minor variations between the models analyzed (null and adjusted), which suggests that the contextual *per capita* income variable contributed very little to explain the variance of the outcome between the levels of analysis.

**Table 3:** Multilevel logistic regression models for association between contextual income and incidence of disability in instrumental activities of daily living (IADL). 2009/2010 and 2013/2014 EpiFloripa Elderly study, Florianópolis, state of Santa Catarina, Brazil.

Variable		Empty model	Model 1	Model 2	Model 3
		OR (95%CI)	OR (95%CI)	OR (95%CI)	OR (95%CI)
Fixed effects					
	Intercept	0,15 (0,12–0,18)	0,16 (0,12–0,22)	0,16 (0,10–0,25)	0,15 (0,09–0,27)
Contextual level					
Contextual *per capita* income (terciles in BRL)					
< 1,101			1,00	1,00	1,00
1,101–1,864			0,67 (0,43–1,07)	0,68 (0,43–1,08)	0,69 (0,43–1,10)
> 1,864			1,02 (0,68–1,54)	1,06 (0,70–1,61)	1,10 (0,70–1,70)
Individual level					
Age (years)				
	60–69			1,00	1,00
	70–79			1,22 (0,82–1,80)	1,22 (0,82–1,81)
	≥ 80			0,65 (0,38–1,12)	0,65 (0,38–1,12)
Sex					
	Male			1,00	1,00
	Female			1,02 (0,70–1,48)	1,00 (0,69–1,47)
Equalized *per capita* income (terciles in BRL)					
	< 1,281.42				1,00
	1,281.72–3,000				0,93 (0,60–1,64)
	> 3,000				0,90 (0,56–1,44)
Random effects					
	ICC (%)	0,40	0,00	0,00	0,00

ICC: intraclass correlation coefficient

## DISCUSSION

In this study, the incidence of disability among sampled seniors was 15.8% in BADL and 13.4% in IADL after a four-year period. Although disability is considered one of the key markers of elderly health, longitudinal data about its occurrence in activities of daily living are still scarce in the literature, especially in populations of medium and low income countries, which hinders the conduct of comparisons. We found two studies with Brazilian elderly. In the Epidoso cohort study (epidemiology of the elderly) in the municipality of São Paulo, the incidence of functional loss in seven or more BADL or IADL was 17.8% after three years^5^. While among the elderly in the SABE study (on health, well-being, and aging), also held in São Paulo, the density of incidence of disability in IADL was 44.7/1,000 persons/year for women and 25.2/1,000 persons/year for men, after six-year follow-up^11^.

International studies, in American seniors, found incidence of 2.5% in BADL^17^ and 48% in IADL^18^ in separate follow-up periods that ranged from three to nine years, respectively. In another study that followed the elderly of the Epese (Established Populations for Epidemiologic Studies of the Elderly) project, the incidence of severe disability in three or more BADL or IADL was 6.8%, after five years^19^. While in the one-year follow-up with long-lived elderly (aged ≥ 90 years), the incidence of disability (16.5%) in activities of both domains was even higher^20^.

In general, despite disparities observed in methodologies of the studies analyzed, especially as to categorization of outcomes and sample follow-up period, the findings indicate a tendency in the development of disability. Proportions rise as age increases, with higher incidences usually observed in IADL than in BADL in younger elderly, especially because performing the first requires fine motor skills and preserved mobility, which tend to be undermined early in the aging process^21^. As for differences between the sexes, we observed higher prevalence of disability among females^22^ ^,^ ^23^; however, incidence is similar in both sexes^12^. This result was also observed in this study, in which the difference in incidence of disability between men and women was minor, especially in IADL, with 12.9% and 13.6%, respectively.

As for influence of contextual neighborhood income on incidence of outcomes, the elderly living in intermediate income tercile had 37% lower chance of developing disability in BADL than those living in the lower tercile, even after adjusting for individual variables. Most published studies that analyzed the association of neighborhood socioeconomic position with presence of disabilities present cross-sectional design^8^ ^,^ ^10^ ^,^ ^24^, which limits direct comparisons. However, the findings in most of these publications are close to this study, in which the elderly evaluated that lived in areas of greater socioeconomic advantage (higher income, educational level, and employment rate) had lower chances of disability in BADL.

Of the two longitudinal studies found^9^ ^,^ ^25^, statistically significant differences were observed only in one, in which Chinese elderly (aged ≥ 65 years) who lived in neighborhoods with higher *per capita* gross domestic product (GDP) value (≥ BRL 2,000) showed higher chances of developing disabilities in BADL than those living in neighborhoods with lower GDP values^9^. However, we consider that such finding differs from that found in this study because, despite significant association was observed only in the middle income category, the chances of occurrence of disability among the elderly of the high tercile were also lower than those of the lower tercile. Therefore, we believe that residing in municipality sectors with higher income appears to reduce the development of disabilities in BADL.

The main theories that support these associations are based mainly on the fact that more economically disadvantaged locations provide less access to and few options of services to the local community, which minimizes the social interaction and support between residents and, thus, contributes to the development of disabilities^8^ ^,^ ^9^. Original studies^5^ ^,^ ^26^ and metanalysis studies^27^ that investigated the effect of social support on disability indicate that performing active leisure activities and socializing with friends and neighbors provide fundamental relations of cooperation and interactivity and help keep some skills preserved, such as memory and spatial attention, considered critical to performing activities of daily living independently. In addition, neighborhoods with greater social deprivation reflect smaller network of contacts and, in some ways, restrict social relations, intensifying the bond with the family and, consequently, the dependency for performance of basic activities of daily life^24^. There is also evidence that participation in community groups increases the access of older persons to information on health prevention and care, as well as provides positive effects on self-esteem and satisfaction with life, encouraging them to keep themselves independent^28^.

It is believed that the absence of association between contextual income and occurrence of disability in IADL may be related to the fact that performance of these tasks depends on characteristics involving the built environment that, although most times are related to socioeconomic conditions of the place, were not directly evaluated in this study. Some of these characteristics include the presence of quality streets and sidewalks and availability of accessible physical spaces, green areas, and leisure spaces that promote active behavior and greater participation in the community^24^ ^,^ ^25^, thus helping in keeping the independence of the elderly in activities outside the home.

The challenges faced by researchers on this subject include the use of contextual data from national surveys, which often do not reflect the actual space of the neighborhood of the researched sample^29^. In this study, the geographical area was delimitated by the census tracts, which is not necessarily equal to the neighborhood's physical space, or to the social interaction area used by the seniors. Another limitation observed in this study as to the use of secondary data refers to the absence of socioeconomic variables, in addition to income, that could be analyzed to represent the context.

However, we present potential that should be highlighted. To our knowledge, this is the first study with longitudinal design conducted in Brazil that sought to investigate the incidence of disabilities in BADL and IADL in a sample representing the elderly population and the influence of living in environments with different income strata on their occurrence. The results can contribute to the planning of local and intersectoral initiatives, with focus on reducing socioeconomic inequities, aiming at income redistribution and, above all, at promotion of social inclusion activities for the elderly population that resides in the lower-income sectors of the municipality.

Another characteristic that should be highlighted refers to the separate analyses of the two outcomes that represent disability (IADL and BADL), which is important, since the loss of independence in these domains tends to occur in different ways as age increases. Neighborhood income having been associated only with BADL shows greater urgency of investments in prevention initiatives, since these basic activities represent disability in its most severe form, being normally affected after IADL. Also concerning the study methodology, it should be noted as a positive point the absence of significant losses of seniors between the two waves according to the variables studied, except for the age variable (p < 0.001), which presented higher loss of seniors aged over 80 years due to deaths in this category^13^.

Based on the results, we conclude that the elderly living in census sectors with intermediate contextual income tercile have lower chances of developing disability in BADL over four years than those of the lower tercile. We consider important that new follow-ups are carried out to estimate the incidence of disabilities among the Brazilian elderly and the influence of living in neighborhoods with different socioeconomic positions on their development, aiming at obtaining data that enable better comparisons and assist in public decision-making concerning the necessary interventions. Finally, we highlight that the country is experiencing a period of great social, political, and economic challenges related to the accelerated increase in the number of elderly individuals and should, therefore, consider this population as priority in strategies involving the promotion of functional independence.
